# Dopamine Receptor Type 2-Expressing Medium Spiny Neurons in the Ventral Lateral Striatum Have a Non-REM Sleep-Induce Function

**DOI:** 10.1523/ENEURO.0327-23.2023

**Published:** 2023-09-26

**Authors:** Tomonobu Kato, Kenji F. Tanaka, Akiyo Natsubori

**Affiliations:** 1 Division of Brain Sciences, Institute for Advanced Medical Research, Keio University School of Medicine, Shinjuku-ku, Tokyo 160–8582, Japan; 2 Sleep Disorders Project, Tokyo Metropolitan Institute of Medical Science, Setagaya-Ku, Tokyo 156-8506, Japan; 3 Faculty of Science and Technology, Keio University, Kohoku-ku, Yokohama, Kanagawa 223–8522, Japan

**Keywords:** dopamine, optogenetics, photometry, population activity, sleep, ventral striatum

## Abstract

Dopamine receptor type 2-expressing medium spiny neurons (D2-MSNs) in the medial part of the ventral striatum (VS) induce non-REM (NREM) sleep from the wake state in animals. However, it is unclear whether D2-MSNs in the lateral part of the VS (VLS), which is anatomically and functionally different from the medial part of the VS, contribute to sleep-wake regulation. This study aims to clarify whether and how D2-MSNs in the VLS are involved in sleep-wake regulation. Our study found that specifically removing D2-MSNs in the VLS led to an increase in wakefulness time in mice during the dark phase using a diphtheria toxin-mediated cell ablation/dysfunction technique. D2-MSN ablation throughout the VS further increased dark phase wakefulness time. These findings suggest that VLS D2-MSNs may induce sleep during the dark phase with the medial part of the VS. Next, our fiber photometric recordings revealed that the population intracellular calcium (Ca^2+^) signal in the VLS D2-MSNs increased during the transition from wake to NREM sleep. The mean Ca^2+^ signal level of VLS D2-MSNs was higher during NREM and REM sleep than during the wake state, supporting their sleep-inducing role. Finally, optogenetic activation of the VLS D2-MSNs during the wake state always induced NREM sleep, demonstrating the causality of VLS D2-MSNs activity with sleep induction. Additionally, activation of the VLS D1-MSNs, counterparts of D2-MSNs, always induced wake from NREM sleep, indicating a wake-promoting role. In conclusion, VLS D2-MSNs could have an NREM sleep-inducing function in coordination with those in the medial VS.

## Significance Statement

The sleep-inducing function of dopamine receptor type 2-expressing medium spiny neurons (D2-MSNs) in the medial part of the ventral striatum (VS) has been previously reported; however, their function in the lateral part of the VS (VLS) has not been elucidated. We demonstrated that the diphtheria toxin-induced ablation of D2-MSNs in the VLS, as well as in the entire VS, increased wakefulness time in mice during the dark phase. VLS D2-MSNs had higher average Ca^2+^ signals during non-REM (NREM) and REM sleep than wake state via fiber photometric recording. Furthermore, optogenetic activation of VLS D2-MSNs during wake state induced NREM sleep in mice. In conclusion, D2-MSNs in the VLS have an NREM sleep-inducing function in coordination with those in the medial VS.

## Introduction

The regulation of sleep-wake behavior involves various neural populations, including the medium spiny neurons that express dopamine receptor type 2 (D2-MSNs) in the striatum (Oishi and Lazarus, 2017). D2-MSNs express receptors for adenosine, one of the sleep-promoting substances (Huang et al., 2014). These neurons are uniformly distributed throughout the striatum (Kemp and Powell, 1971); however, its functional manifestation is divided between the dorsal striatum and ventral striatum (DS and VS), and further varies depending on the subregion within the DS/VS, such as during reward processing (Cox and Witten, 2019). Thus, the sleep-wake regulatory function of these neurons could also be exhibited differently in each striatal subregion.

In the DS, a region primarily involved in motor control in animals, D2-MSNs contribute to non-REM (NREM) sleep induction in the rostral, centromedial, and centrolateral subregions, but not in the caudal subregion, and their sleep-inducing role is exhibited only in the dark phase (Yuan et al., 2017). In contrast, in the VS, it is not clear in all subregions whether the D2-MSNs are involved in sleep-wake control. The VS can be anatomically and functionally divided into at least three subregions: the nucleus accumbens (NAc) medial shell, referred to as the ventral medial striatum (VMS), NAc core, and ventral lateral striatum (VLS; also known as NAc lateral shell). Previous studies have reported that D2-MSNs in the NAc core subregion contribute to NREM sleep induction in mice (Oishi et al., 2017; Luo et al., 2018). Another study has suggested that these neurons in the NAc medial shell have the same function (Satoh et al., 1999), and mediates caffeine-induced arousal via the adenosine A_2A_ receptor which co-express with dopamine D2 receptor (Lazarus et al., 2011). However, it is unclear whether D2-MSNs in the VLS subregion play a role in regulating sleep-wake cycles in animals. The VLS D2-MSNs receive distinctive input from cortical glutamatergic neurons in the insular cortex and dopaminergic neurons in the ventral tegmental area (VTA; Hunnicutt et al., 2016; Mingote et al., 2019), and specifically constitute neuronal circuitry with parvalbumin-expressing interneurons (Yoshida et al., 2020). This could result in distinct firing patterns and functions in reward processing compared with those in the medial part of the VS (Tsutsui-Kimura et al., 2017a; Yang et al., 2018; R. Chen et al., 2021). This anatomic and functional uniqueness of the VLS predicts a distinct role for VLS D2-MSNs in sleep-wake control.

To elucidate the role of VLS D2-MSNs in sleep-wake regulation, we evaluated the sleep-wake architecture using a 24 h polysomnogram in mice, in which the D2-MSNs were spatiotemporally progressively ablated from the specific VLS to the entire VS based on DOX-dependent diphtheria toxin induction in these neurons (D2-DTA; Tsutsui-Kimura et al., 2017b). Next, we investigated the Ca^2+^ signal patterns of the D2-MSNs in the VLS during physiological sleep-wake states in mice. Furthermore, we conducted optogenetic activation of the VLS D2-MSNs to reveal a causal relationship between the activity of these neurons and sleep-wake regulation. Our findings provide evidence that D2-MSNs in the VLS are involved in sleep induction in animals, in cooperation with those in the medial part of the VS.

## Materials and Methods

### Ethics statement

All animal procedures were conducted following the National Institutes of Health *Guide for the Care and Use of Laboratory Animals* and were approved by the Keio University Animal Experiment Committee in compliance with the Keio University Institutional Animal Care and Use Committee (approval numbers A2022-315).

### Animals

Experiments were conducted with 8- to 14-month-old male and female mice. All mice were maintained on a 12/12 h light/dark cycle (lights on at 8 A.M.; luminous flux, 120 lm). Polysomnographic recordings were performed on all days [start time at 8 A.M. (ZT0)], fiber photometric recordings were performed during the light and dark phases (ZT0–ZT8 and ZT12–ZT20), and optogenetic manipulation was performed during the light and dark phases (ZT0–ZT8 and ZT12–Z16). *Drd2*-DTA mice (*Drd2*-tTA::tetO-DTA [diphtheria toxin A]; hereafter referred to as D2-DTA) were obtained by crossing *Drd2*-tTA mice (Tsutsui-Kimura et al., 2017b) and tetO-DTA mice (Lee et al., 1998). D2-DTA mice were fed doxycycline (DOX)-containing chow until the start of the experiment. Upon switching to normal chow (DOX-off day 0), tTA-mediated DTA induction was initiated as previously described (Tsutsui-Kimura et al., 2017b). D2-YC mice (*Drd2*-tTA::tetO-YCnano50 double-transgenic mice) were generated by crossing *Drd2*-tTA and tetO-YCnano50 mice (Kanemaru et al., 2014). D2-ChR2 mice (*Drd2*-tTA::tetO-ChR2(C128S)-EYFP double transgenic mice) were generated by crossing *Drd2*-tTA and tetO-ChR2 mice (Tanaka et al., 2012). D1-ChR2 mice (*Pde10a2*-tTA::tetO- ChR2(C128S)-EYFP; *Adora2a*-Cre triple-transgenic mice) were obtained by crossing *Pde10a2*-tTA mice, tetO- ChR2(C128S)-EYFP mice, and *Adora2a*-Cre mice. *Pde10a2*-tTA induced the tTA-mediated ChR2(C128S)-EYFP in both D1-MSNs and D2-MSNs; however, D2-MSN-specific Cre expression under the control of an *Adora2a* promoter removed the tetO cassette, resulting in D1-MSNs-specific ChR2(C128S)-EYFP expression (Natsubori et al., 2017). The genetic backgrounds of all transgenic mice were mixed C57BL6 and 129 SvEvTac.

### Surgical procedure

Surgeries were performed using a stereotaxic apparatus (SM-6 M-HT and SM-15R/L, Narishige). Mice were anesthetized with a mixture of ketamine and xylazine (100 and 10 mg/kg, respectively). Body temperature during surgery was maintained at 37 ± 0.5°C using a heating pad (FHC-MO, Muromachi Kikai). Mice received permanent electroencephalography (EEG) and electromyography (EMG) electrodes for polysomnography. Three pits were drilled into the skull using a carbide cutter (drill diameter: 0.8 mm). We implanted two electrodes in each subject, each consisting of a 1.0 mm diameter stainless steel screw that served as an EEG electrode. One implant was placed over the right frontal cortical area (AP: +1.0 mm; ML: +1.5 mm) as a reference electrode, while the other was placed over the right parietal area (AP: +1.0 mm anterior to λ; ML: +1.5 mm) as a signal electrode. Another electrode was placed over the right cerebellar cortex (AP: −1.0 mm posterior to λ; ML: +1.5 mm) as the ground electrode. Two silver wires (AS633; Cooner Wire Company) were placed bilaterally into the trapezius muscles and served as the EMG electrodes. An optic fiber was inserted into the VLS at the following coordinates relative to bregma: AP, +1.1 mm; ML, +1.8 mm; DV, +4.4 mm from the skull unilaterally for photometry, AP, +1.1 mm; ML, ±1.9 mm; DV, +3.1 mm from the brain surface bilaterally for optogenetic manipulation. Finally, the electrode and optical fiber cannula assembly were anchored and fixed to the skull using SuperBond (Sun Medical Co).

### EEG/EMG recordings

The EEG/EMG signals were amplified (gain 1000×) and filtered (EEG: 1–300 Hz, EMG: 10–300 Hz) using a DC/AC differential amplifier (AM-3000, AM Systems). The input was received via an input module (NI-9215, National Instruments), digitized at a sampling rate of 1000 Hz using a data acquisition module (cDAQ-9174, National Instruments), and recorded using a custom-made LabVIEW program (National Instruments). We habituated the 24 h EEG/EMG recordings more than three times, and REM sleep (see the vigilance state assessment) was often observed when we started the experiment. Three days before the control recordings on DOX-off day −1 (DOX-off day −4), the mice were placed in a soundproof box and connected to the EEG/EMG cable for the 24 h EEG/EMG recording of D2-DTA mice. They remained continuously tethered to the cable in the same soundproof box until day 11 of the DOX-off experiment. Cages and food were not changed during the stay in the soundproof box, except changing from DOX-containing chow to normal chow on Dox-off day 0.

### Fiber photometry

The ratiometric fiber photometry method has been described previously (Natsubori et al., 2017; Kato et al., 2022). An excitation light (435 nm; silver light-emitting diode, Prizmatix) was reflected off a dichroic mirror (DM455CFP, Olympus), focused with a 2× objective lens (numerical aperture 0.39, Olympus) and coupled into an optical fiber (400-mm diameter, 0.39 numerical aperture; catalog #M79L01, Thorlabs) through a pinhole (diameter 400 μm). The light-emitting diode power was <200 μW at the fiber tip. The cyan and yellow fluorescence emitted by the YC-nano50 was collected via an optical fiber cannula, divided by a dichroic mirror (DM515YFP, Olympus) into cyan (483/32 nm band path filters, Semrock) and yellow (542/27 nm), and detected using a photomultiplier tube (H10722-210, Hamamatsu Photonics). The fluorescence signals were digitized using a data acquisition module (cDAQ-9174, National Instruments) and recorded simultaneously using a custom-made LabVIEW program (National Instruments). Signals were collected at a sampling frequency of 1000 Hz.

### Optogenetic manipulation

An optical fiber (numerical aperture 0.39, Thorlabs) was inserted through the guide cannula. Blue (470 nm) and yellow (575 nm) light were generated using a SPECTRA 2-LCR-XA light engine (Lumencor). The blue and yellow light power intensities at the tip of the optical fiber were 1–2 and 3–4 mW, respectively. Using EEG and EMG monitoring, we illuminated 1 s of blue light to open the step-function-type opsin ChR2(C128S; Berndt et al., 2009) of D2-ChR2 or D1-ChR2(C128S) mice during the wake or NREM state lasting >10 s, respectively. In control trials, yellow light was used instead of blue light.

### Mouse vigilance state assessment

EEG/EMG signals were analyzed using MATLAB (MathWorks). Power spectral EEG data were obtained using multitaper spectral estimation (McCoy et al., 1998). A power spectral profile over a 1- to 50-Hz window was used for the analysis. We detected each sleep-wake state scored offline by visually characterizing 10-s epochs for 24-h polysomnography of D2-DTA mice and 1-s epochs for fiber photometric recording of D2-YC mice and optogenetic experiments with D2-ChR2 and D1-ChR2 mice (Funato et al., 2016; Kato et al., 2022). The state determination criteria were as follows: the wake state was characterized by a low-amplitude fast EEG and high-amplitude variable EMG. NREM sleep is characterized by a high-amplitude δ (1–4 Hz) frequency EEG and low-amplitude tonus EMG. REM sleep was staged using θ (6–9 Hz) dominant EEG and electromyography (EMG) atonia (Tobler et al., 1997).

### Colorimetric *in situ* hybridization (ISH)

Mice were deeply anesthetized with ketamine (100 mg/kg) and xylazine (10 mg/kg) and perfused with a 4% paraformaldehyde phosphate buffer solution. After removing the brains from the skull, they were fixed overnight in the same solution. The brains were then cryoprotected in 20% sucrose overnight, frozen, and sectioned to 25-μm thickness on a cryostat. The sections were mounted on silane-coated glass slides (Matsunami Glass), and then treated with proteinase K (40 μg ml^−1^; Merck). After washing and acetylation, sections were incubated with digoxigenin (DIG)-labeled complementary RNA (cRNA) probes. After the sections were washed in buffers with serial differences in stringency, they were incubated with an alkaline phosphatase-conjugated anti-DIG antibody (11093274910, 1:5000; Roche). The cRNA probes were visualized using freshly prepared colorimetric substrate (NBT/BCIP; Roche). Nuclear Fast Red (Vector Laboratories) was used for counterstaining (Tanaka et al., 2012). Probes for *DTA*, *Drd1*, and *Drd2* were used in this study. Images were obtained using an all-in-one microscope (BZ-X710; Keyence).

### Data processing

All animals and trials were randomly assigned to experimental conditions. The experimenters were not blinded to the experimental conditions during data collection and analysis. Mice were excluded when the optical fiber position was incorrectly targeted. We used custom-made programs in MATLAB for signal processing.

### EEG/EMG signal analysis

The EEG signals were bandpass (FIR)–filtered (Kaiser window). The EEG frequency bands were set as follows: δ: 1–4 Hz; θ: 6–9 Hz; σ: 9–12 Hz; β: 12–30 Hz; and γ: 30–50 Hz (Choi et al., 2010). The power of each EEG frequency band was obtained using the square of the amplitude.

### Fluorescence signal analysis

For fiber photometric signal analysis, the yellow and cyan fluorescence signals were detrended. Further, we used the YC ratio (*R*), which is the ratio of yellow to cyan fluorescence intensity, calculated as the population Ca^2+^ signals, and then Z-scored. For the power spectral analysis, fiber photometric signals (YC signals) were extracted during the uninterrupted wake, NREM sleep, and REM sleep bouts that lasted >100 s, high pass filtered with a 0.01-Hz cutoff frequency, and calculated with a wavelet transform using a Morse wavelet from 0.01 to 1 Hz with time-bandwidth parameter (*P*^2^ = 10). The peak frequency is determined as the local maximum of the power spectrum. For the state transition analysis, YC signals were extracted during uninterrupted wake, NREM sleep, and REM sleep bouts that lasted >20 s, and normalized using the mean and SD of YC signals from 20 s before the transition to the transition point.

### Statistical analysis

All experimental data were analyzed using the following parametric statistics: Student’s *t* test (independent samples *t* test), paired *t* test, paired *t* test with Bonferroni correction, and repeated-measures ANOVA followed by the Tukey–Kramer *post hoc* test.

### Data availability

The datasets generated and/or analyzed in the current study are available from the corresponding author on reasonable request.

## Results

### Ablation of D2-MSNs in the VLS causes an increase in wakefulness time in the dark phase

**Figure 1.**
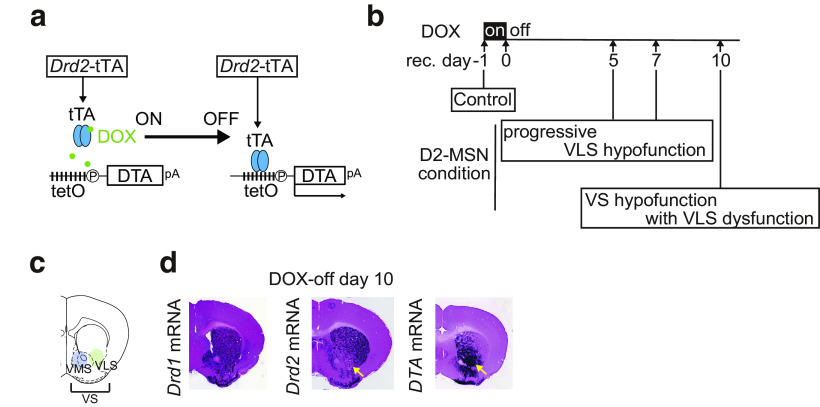
Spatiotemporally specific the VS D2-MSNs ablation. ***a***, DOX-controllable DTA expression. *Drd2*-tTA::tetO-DTA (D2-DTA) mice were fed with doxycycline (DOX)-containing chow until the start of the experiment (DOX-on). *DTA* mRNA expression started when DOX-chow was replaced with normal chow (DOX-off). ***b***, Time course of the VS D2-MSNs ablation, and timing of the polysomnographic recordings. ***c***, Schematic illustration of the VS, including the VMS and the VLS. ***d***, *Drd1*, *Drd2*, and *DTA* mRNA levels in the striatum on DOX-off day 10. Purple denotes the mRNA signal. Left, *Drd1* mRNA expression throughout the striatum. Middle, *Drd2* mRNA expression in the VLS disappears (yellow arrow). Right, *DTA* mRNA expression was observed throughout the VS (yellow arrow).

To investigate the role of D2-MSNs in the sleep-wake regulation within the ventral lateral striatum (VLS), we conducted repeated 24-h polysomnography on *Drd2*-tTA::tetO-DTA mice (D2-DTA; Tsutsui-Kimura et al., 2017b). In this mouse line, tTA-mediated diphtheria toxin expression is induced in D2-MSNs, which is initiated specifically in the VLS and follows spatial progress within the ventral striatum (VS) in doxycycline (DOX)-dependent manner (Fig. 1*a*): upon switching to normal chow from DOX-containing chow (DOX-off day 0), DTA induction is first observed only in the VLS (corresponding to the NAc lateral shell) on DOX-off day 3, and the *DTA* mRNA-positive cells were increased and the induction area gradually expands toward the medial part of the VS (corresponding to the NAc medial shell and core) with each passing day (Tsutsui-Kimura et al., 2017b). Furthermore, based on the altered electrophysiological functions in viable DTA-expressing VLS D2-MSNs (Tsutsui-Kimura et al., 2017b), the VLS D2-MSNs could be regarded as a progressive hypofunction condition on DOX-off day 5 and day 7, respectively (Fig. 1*b*). On DOX-off day 10, *DTA* mRNA was expressed in the entire VS with loss of *Drd2* mRNA expression in the VLS, indicating that the D2-MSNs in the entire VS could be in a hypofunctional state with VLS D2-MSNs dysfunction (Fig. 1*c*,*d*).

**Figure 2.**
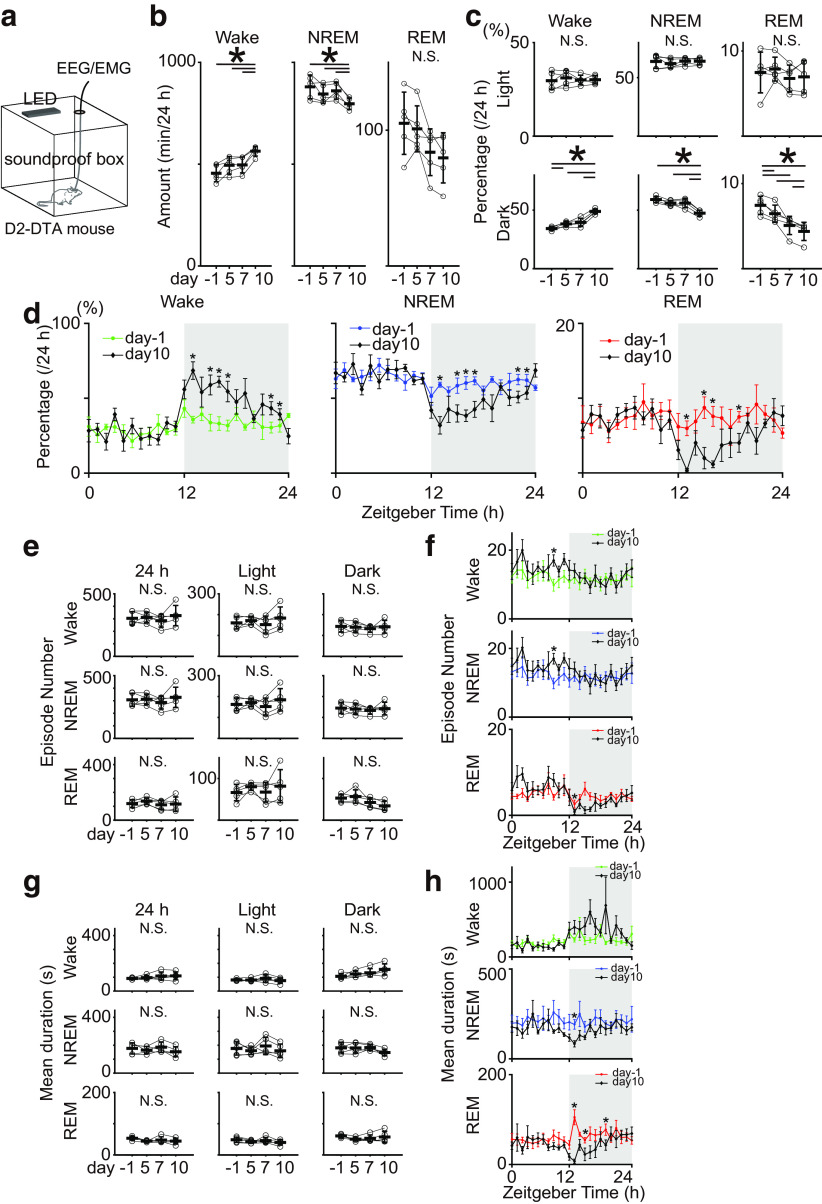
Analysis of sleep structure before and after the VLS/VS D2-MSNs ablation. ***a***, Experimental setup. We conducted 24 h EEG/EMG recording in a soundproof box and set a 12/12 h light/dark cycle using LED light. ***b***, Total amount of wake, NREM sleep, and REM sleep time in control (DOX-off day −1) and DOX-off days 5, 7, and 10 (*n* = 5 mice). Repeated-measures ANOVA was followed by the Tukey–Kramer *post hoc* test (Results of statistical tests is shown in Table 1). ***c***, Time spent in each sleep state during the light and dark phases in control (DOX-off day −1) and DOX-off mice on days 5, 7, and 10 (*n* = 5 mice). Repeated-measures ANOVA was followed by the Tukey–Kramer *post hoc* test (Results of statistical tests is shown in Table 2). ***d***, Daily variations in wake time (left), NREM sleep (middle), REM sleep (right) every hour in control (DOX-off day −1), and DOX-off day 10 (*n* = 5 mice). Two-way repeated-measures ANOVA was followed by the Tukey–Kramer *post hoc* test (Results of statistical tests is shown in Table 3). ***e***, Episode numbers of wake, NREM sleep, and REM sleep for 24 h, light, and dark phases in control (DOX-off day −1) and DOX-off days 5, 7, and 10 (*n* = 5 mice). Repeated-measures ANOVA. Wake (24 h): *p *=* *0.62; wake (light), *p *=* *0.36; wake (dark), *p *=* *0.89; NREM (24 h): *p *=* *0.60; NREM (light), *p *=* *0.33; NREM (dark), *p *=* *0.88; REM (24 h): *p *=* *0.30; REM (light), *p *=* *0.42; REM (dark), *p *=* *0.88. ***f***, Daily variations in number of episodes during each sleep/wake state in control (DOX-off day −1), DOX-off day 10 (*n* = 5 mice). Two-way repeated-measures ANOVA was followed by the Tukey–Kramer *post hoc* test). Results of statistical tests is shown in Table 4. ***g***, Mean duration of each bout of wake, NREM sleep, and REM sleep for 24 h, light, and dark phases in control (DOX-off day −1) and DOX-off days 5, 7, and 10 (*n* = 5 mice). Repeated-measures ANOVA. Wake (24 h): *p *=* *0.21, Wake (light): *p *=* *0.22, Wake (dark): *p *=* *0.04 (no significant difference between each group using the Tukey–Kramer *post hoc* test), NREM (24 h): *p *=* *0.20, NREM (light): *p *=* *0.29, NREM (dark): *p *=* *0.16, REM (24 h): *p *=* *0.14, REM (light): *p *=* *0.37, REM (dark): *p *=* *0.16. ***h***, Daily variations in mean duration during each sleep-wake state in control (DOX-off day −1) and DOX-off day 10 (*n* = 5 mice). Two-way repeated-measures ANOVA was followed by the Tukey–Kramer *post hoc* test). Results of statistical tests is shown in Table 5. Error bars indicate SEM; *p < 0.05.

We performed 24-h polysomnography recordings to evaluate the effect of progressive ablation of the VS D2-MSNs in the D2-DTA mice on the sleep-wake architecture (Fig. 2*a*). The recordings were done on DOX-off day −1 (control), DOX-off days 5 and 7 (functional ablation of VLS D2-MSNs), and DOX-off day 10 (ablation of D2-MSNs in the entire VS) to assess the impact of these ablations (Fig. 1*b*). We observed that the daily amount of wakefulness time significantly increased on DOX-off day 10, alongside a decrease in NREM sleep time, compared with those on DOX-off day −1 (*p <* *0.05, Repeated-measures ANOVA followed by the Tukey–Kramer *post hoc* test**;** Fig. 2*b*). However, the daily sleep-wake time did not significantly change on DOX-off days 5 and 7. Next, we separately analyzed the effect of DOX-dependent D2-MSNs ablation on sleep-wake architecture in the light and dark phases. In the light phase, there were no significant differences in the percentage of time spent in the wake, in NREM sleep, or REM sleep (Fig. 2*c*). During the dark phase, we observed a significant increase in the percentage of time spent in the wake on DOX-off day 5, which continued to increase up to DOX-off day 10, whereas those in the NREM and REM sleep decreased on the DOX-off day 10 (*p <* *0.05, repeated-measures ANOVA followed by the Tukey–Kramer *post hoc* test; Fig. 2*c*). This phenotype of increased wake time and decreased sleep time because of D2-MSNs ablation was most pronounced in the first half and end of the dark phase (*p <* *0.05, two-way repeated-measures ANOVA was followed by the Tukey–Kramer *post hoc* test; Fig. 2*d*).

**Table 1:** Results of Tukey–Kramer *post hoc* test for daily amount of sleep/wake times among DOX-off days for the data in Figure 2*b*

		Total wake time	Total NREMS time	Total REMS time
Comparison		*p* value	*p* value	*p* value
DOX-off day −1 vs	Day 5	0.17	0.33	0.86
	Day 7	0.29	0.83	0.14
	Day 10	0.006*	0.03*	0.16
DOX-off day 5 vs	Day −1	-	-	-
	Day 7	0.99	0.47	0.14
	Day 10	0.008*	0.03*	0.20
DOX-off day 7 vs	Day −1	-	-	-
	Day 5	-	-	-
	Day 10	0.008*	0.007*	0.60

* *p *<* *0.05.

**Table 2:** Results of Tukey–Kramer *post hoc* test for daily percentage of sleep/wake times in light/dark phases among DOX-off days for the data in Figure 2*c*

		Light phase			Dark phase		
		Total wake percentage	Total NREMS percentage	Total REMS percentage	Total wake percentage	Total NREMS percentage	Total REMS percentage
Comparison		*p* value	*p* value	*p* value	*p* value	*p* value	*p* value
DOX-off day −1 vs	Day 5	0.71	0.77	0.92	0.005*	0.13	0.03*
	Day 7	0.99	1	0.77	0.14	0.53	0.008*
	Day 10	0.98	1	0.94	0.001*	0.006*	0.007*
DOX-off day 5 vs	Day −1	-	-	-	-	-	-
	Day 7	0.78	0.37	0.18	0.7	1	0.12
	Day 10	0.92	0.61	0.69	0.0004*	0.003*	0.047*
DOX-off day 7 vs	Day −1	-	-	-	-	-	-
	Day 5	-	-	-	-	-	-
	Day 10	1	1	0.98	0.0005*	0.0004*	0.01*

* *p *<* *0.05.

**Table 3:** Results of Tukey–Kramer *post hoc* test for daily variations of sleep/wake times between before and after the DOX-off for the data in Figure 2*d*

	DOX-off day −1 vs day 10		
	*p* value		
Time of day	Wake	NREMS	REMS
ZT1	0.81	0.67	0.71
ZT2	0.68	0.55	0.47
ZT3	0.21	0.22	0.99
ZT4	0.25	0.18	0.94
ZT5	0.22	0.33	0.29
ZT6	0.31	0.27	0.19
ZT7	0.57	0.66	0.71
ZT8	0.84	0.61	0.27
ZT9	0.33	0.23	0.75
ZT10	0.10	0.22	0.41
ZT11	0.63	0.93	0.30
ZT12	0.16	0.29	0.12
ZT13	0.001*	0.004*	0.003*
ZT14	0.29	0.33	0.28
ZT15	0.002*	0.003*	0.01*
ZT16	0.02*	0.04*	0.008*
ZT17	0.03*	0.02*	0.31
ZT18	0.29	0.38	0.17
ZT19	0.005*	0.07	0.04*
ZT20	0.91	0.93	0.60
ZT21	0.35	0.36	0.32
ZT22	0.003*	0.006*	0.40
ZT23	0.01*	0.04*	0.70
ZT24	0.08	0.08	0.15

* *p *<* *0.05. ZT: zeitgeber time.

**Table 4:** Results of Tukey–Kramer *post hoc* test for daily variations of sleep/wake episode numbers between before and after the DOX-off for the data in Figure 2*f*

	DOX-off day −1 vs day 10		
	*p* value		
Time of day	Wake	NREMS	REMS
ZT1	0.74	0.70	0.48
ZT2	0.53	0.48	0.11
ZT3	0.25	0.30	0.07
ZT4	0.48	0.57	0.51
ZT5	0.84	0.64	0.88
ZT6	0.18	0.37	1
ZT7	0.92	0.92	0.66
ZT8	0.74	0.73	0.73
ZT9	0.04*	0.03*	0.12
ZT10	0.41	0.39	1
ZT11	0.12	0.13	0.88
ZT12	0.32	0.29	0.80
ZT13	0.51	0.46	0.04*
ZT14	0.51	0.55	0.34
ZT15	0.77	0.78	0.05
ZT16	0.49	0.44	0.06
ZT17	0.72	0.70	0.60
ZT18	0.95	1	0.32
ZT19	0.30	0.48	0.39
ZT20	0.52	0.62	0.30
ZT21	0.55	0.50	0.38
ZT22	0.89	0.81	0.57
ZT23	0.84	0.84	0.82
ZT24	0.61	0.64	0.52

* *p *<* *0.05. ZT: zeitgeber time.

**Table 5:** Results of Tukey–Kramer *post hoc* test for daily variations of mean durations of sleep-wake states between before and after the DOX-off for the data in Figure 2*h*

	DOX-off day −1 vs day 10		
	*p* value		
Time of day	Wake	NREMS	REMS
ZT1	0.55	0.75	0.44
ZT2	0.55	0.54	0.91
ZT3	0.22	0.29	0.18
ZT4	0.85	0.16	0.70
ZT5	0.19	0.67	0.72
ZT6	0.59	0.17	0.55
ZT7	0.63	0.70	0.95
ZT8	0.60	0.86	0.30
ZT9	0.07	0.18	0.19
ZT10	0.76	0.18	0.14
ZT11	0.13	0.18	0.34
ZT12	0.76	0.16	0.06
ZT13	0.13	0.02*	0.004*
ZT14	0.98	0.17	0.19
ZT15	0.10	0.15	0.02*
ZT16	0.11	0.83	0.14
ZT17	0.29	0.07	0.31
ZT18	0.84	0.64	0.68
ZT19	0.24	0.50	0.03*
ZT20	0.69	0.84	0.43
ZT21	0.27	0.95	0.51
ZT22	0.18	0.33	0.69
ZT23	0.62	0.51	0.27
ZT24	0.31	0.63	0.52

* *p *<* *0.05. ZT: zeitgeber time.

**Figure 3.**
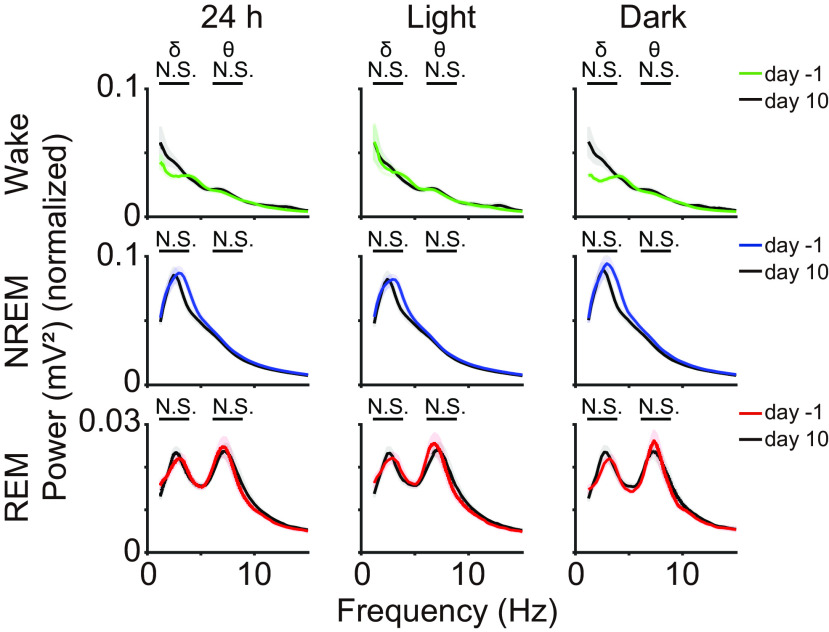
Comparison of EEG power spectrum before and after the VS D2-MSNs ablation. EEG spectra before (DOX-off day −1) and after (DOX-off day 10) the VS D2-MSNs ablation during each sleep-wake state (*n* = 5 mice). Two-way repeated-measures ANOVA was followed by the Tukey–Kramer *post hoc* test). Results of statistical tests is shown in Table 6. Colored shades indicate SEM; *p <* *0.05.

**Table 6:** Results of Tukey–Kramer *post hoc* test for each EEG band power between before and after the DOX-off for the data in **Figure 3**

	Wake			NREMS			REMS		
	DOX-off day −1 vs day 10			DOX-off day −1 vs day 10			DOX-off day −1 vs day 10		
	*p* value			*p* value			*p* value		
EEG band power	24 h	Light	Dark	24 h	Light	Dark	24 h	Light	Dark
Δ	0.30	0.75	0.32	0.14	0.20	0.16	0.96	0.51	0.48
θ	0.12	0.42	0.36	0.16	0.29	0.08	0.98	0.98	0.98
σ	0.80	0.15	0.80	0.10	0.11	0.71	0.27	0.26	0.34
β	0.42	0.35	0.43	0.15	0.16	0.13	0.12	0.08	0.39
γ	0.99	0.70	0.83	0.85	0.88	0.54	0.33	0.33	0.36

* *p *<* *0.05.

To determine the characteristics of sleep-wake alteration induced by D2-MSNs ablation, we calculated the number of episodes of wake, NREM sleep, and REM sleep. Although the mean duration of the sleep/wake bout showed a gradual increasing trend in the dark phase under progressive D2-MSNs ablation, they were almost unaffected (*p <* *0.05, repeated-measures ANOVA followed by the Tukey–Kramer *post hoc* test; Fig. 2*e*, paired *t* test at each time point with Bonferroni correction; Fig. 2*f*). The mean duration of the sleep/wake bout showed a gradual increasing trend in the dark phase under progressive D2-MSNs ablation, although this was not statistically significant (*p <* *0.05, Repeated-measures ANOVA followed by the Tukey–Kramer *post hoc* test; Fig. 2*g*, two-way repeated-measures ANOVA was followed by the Tukey–Kramer *post hoc* test; Fig. 2*h*).

Next, we investigated the EEG changes in sleep or wakefulness that were altered by VS D2-MSN ablation. When we compared the EEG spectrum during each sleep-wake state throughout the day and in the light/dark phases between the control (DOX-off day −1) and DOX-off day 10, there were no significant differences in any of the power bands of the EEG signal (δ, θ, σ, β, and γ; *p <* *0.05, paired *t* test; δ and θ ranges are shown in Fig. 3).

### VLS D2-MSNs display higher Ca^2+^ signal levels during NREM and REM sleep compared with wake

**Figure 4.**
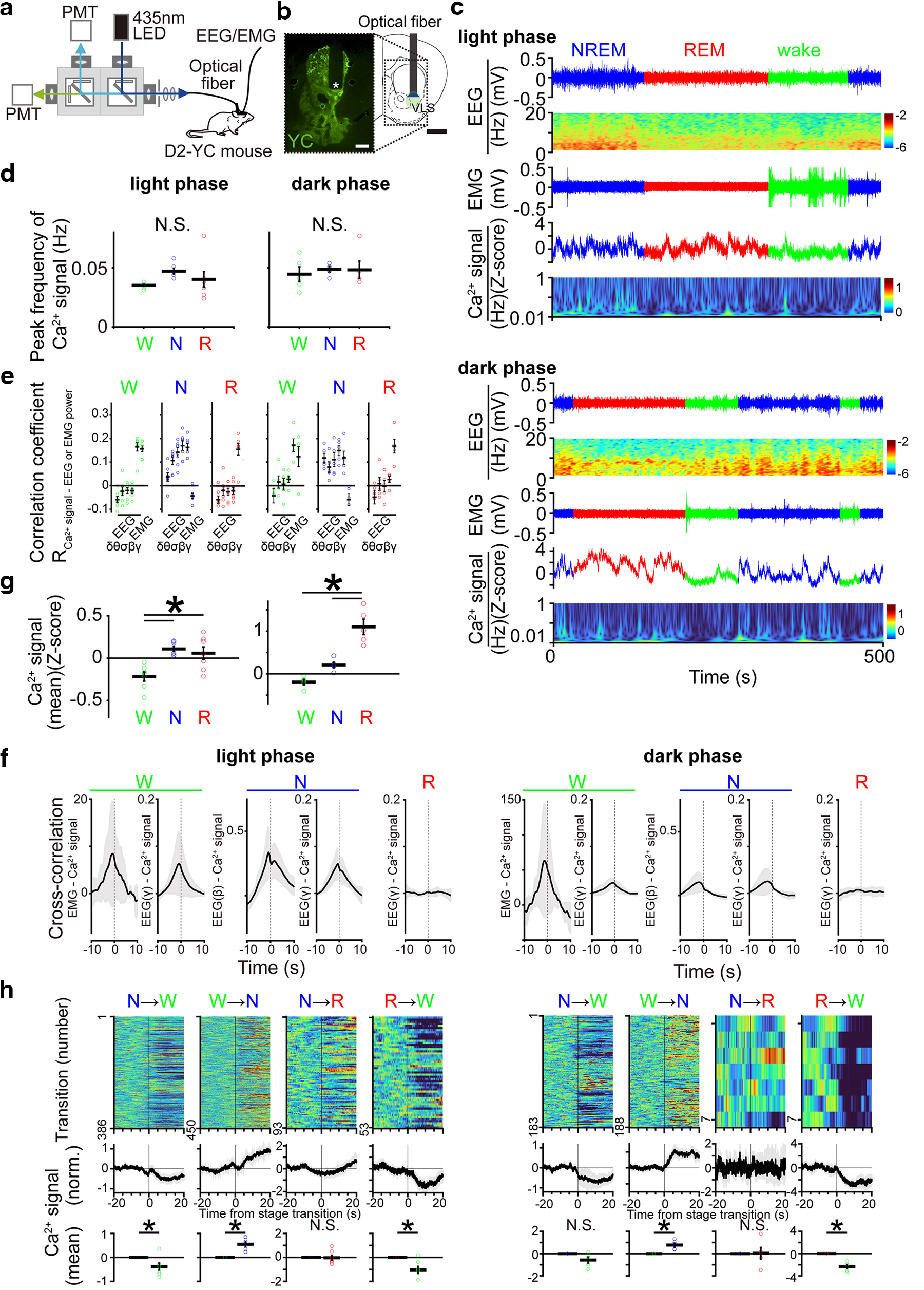
Population Ca^2+^ signals of the VLS D2-MSNs across the sleep-wake states. ***a***, Schematic illustration of the fiber photometry system for monitoring intracellular Ca^2+^ signals of VLS D2-MSNs in D2-YC mice. The EEG and EMG were recorded simultaneously. PMT, photomultiplier tube. ***b***, YCnano50 fluorescent expression in D2-YC mice (left) and a schematic of the fiber photometric recording site (right) in the VLS. The asterisks indicate the tip of the optical fiber. Scale bar, 1 mm. ***c***, Representative examples of EEG signal trace and power spectrogram, EMG signal, and population Ca^2+^ signal trace and spectrogram of VLS D2-MSNs across the sleep-wake states during the light (upper panel) and dark (lower panel) phases. The green line shows wakefulness, the blue line shows NREM sleep and the red line shows REM sleep. ***d***, Peak frequency of Ca^2+^ signals in VLS D2-MSNs during each sleep-wake state during the light (left)/dark (right) phases (28 recordings from 7 mice and 11 recordings from 5 mice, respectively; one-way ANOVA followed by Tukey–Kramer *post hoc* test, wake vs NREM, *p *=* *0.13 and *p *=* *0.37, respectively; NREM vs REM, *p *=* *0.47 and *p *=* *0.72, respectively; wake vs REM, *p *=* *0.67 and *p *=* *0.82, respectively). ***e***, Correlation coefficient between the Ca^2+^ signals and each frequency band (δ, θ, σ, β, and γ) of EEG power or EMG power during wake and NREM sleep, each frequency band (δ, θ, σ, β, and γ) of EEG power during REM sleep during light (left)/dark (right) phases. ***f***, Cross-correlation between EMG power and Ca^2+^ signals during wake, between EEG γ power and Ca^2+^ signals during wake, between EEG β and γ power and Ca^2+^ signals during NREM sleep, and between EEG γ power and Ca^2+^ signals during REM sleep during light (left)/dark (right) phases. ***g***, Mean Ca^2+^ signal levels of the VLS D2-MSNs across the sleep-wake states during light (left)/(right) phases (28 recordings from 7 mice and 11 recordings from 5 mice, respectively; one-way ANOVA followed by Tukey–Kramer *post hoc* test, wake vs NREM, *p *=* *1.7 × 10^−3^ and *p *=* *0.08, respectively; NREM vs REM, *p *=* *0.80 and *p *=* *1.2 × 10^−5^, respectively; wake vs REM, *p *=* *7.0 × 10^−3^ and *p *=* *4.1 × 10^−4^, respectively). ***h***, The Ca^2+^ signal of the VLS D2-MSNs aligned with each sleep/wake transition during light (left)/dark (right) phases. Upper panel, Ca^2+^ signal fluctuations during individual transitions with color-coded fluorescence intensity (NREM to wake, *n* = 386 and 183; wake to NREM, *n* = 450 and 188; NREM to REM, *n* = 93 and 7; REM to wake, *n* = 53 events and 7 from 7 and 5 mice, respectively). Middle panel, Average Ca^2+^ signals from all transitions. Lower panel, Mean of Ca^2+^ signal levels before and after 20 s from each stage transition (VLS: *p*_NREM-wake_ = 3.7 × 10^−3^ and *p *=* *0.10, respectively, *p*_wake-NREM_ = 0.03 and *p *=* *0.01, respectively, *p*_NREM-REM_ = 0.33 and *p *=* *0.96, respectively, *p*_REM-wake_ = 0.02 and *p *=* *3.3 × 10^−3^, respectively; paired *t* test). Gray shading and error bars indicate SEM; *p <* *0.05. W, wake; N, NREM sleep; R, REM sleep.

Our ablation study revealed that D2-MSNs in the VLS may be involved in sleep-wake regulation. Thus, we monitored the compound intracellular calcium (Ca^2+^) signal patterns of the VLS D2-MSNs to determine their activity patterns across sleep-wake states and during sleep/wake stage transitions in mice. We used a fiber photometry system to monitor intracellular Ca^2+^ signals from the VLS D2-MSNs in freely moving mice (Fig. 4*a*,*b*). We used transgenic mice expressing the FRET-based ratiometric Ca^2+^ indicator YC-nano50 (Horikawa et al., 2010), in D2-MSNs under the control of the *Drd2* promoter (*Drd2*-tTA::tetO-YCnano50: D2-YC mice; Natsubori et al., 2017). The ratio of yellow to cyan fluorescence intensity (YC ratio) represented the intracellular Ca^2+^ signal of D2-MSNs.

We found that the VLS D2-MSNs displayed infra-slow periodic Ca^2+^ signal fluctuations during wake, NREM sleep, and REM sleep in both the light and the dark phases (Fig. 4*c*). The peak frequency of these events did not show a significant variation between the different sleep-wake states both in the light and the dark phases (Fig. 4*d*). Next, we investigated the temporal relationship between Ca^2+^ signal fluctuations in the VLS D2-MSNs and EEG/EMG activity during each sleep-wake state. When we calculated the correlation coefficient between the Ca^2+^ signal and the EEG or EMG power, the Ca^2+^ signal in the VLS D2-MSNs shows positive correlation with EEG γ power (correlation coefficient: 0.16 ± 0.02 and 0.17 ± 0.03 in the light and dark phases, respectively; mean ± SEM) and EMG power during the wake (correlation coefficient: 0.16 ± 0.01 and 0.12 ± 0.04, respectively; mean ± SEM), EEG β (correlation coefficient: 0.17 ± 0.02 and 0.15 ± 0.02, respectively; mean ± SEM) and γ power (0.16 ± 0.02 and 0.12 ± 0.03, respectively; mean ± SEM) during the NREM sleep, and EEG γ power during the REM sleep (0.15 ± 0.02 and 0.17 ± 0.03, respectively; mean ± SEM; Fig. 4*e*). Using cross-correlation analysis, we found that the Ca^2+^ signal in VLS D2-MSNs preceded the peak of EEG high-frequency or EMG power during both wake (lag time between peaks, EEG γ power – Ca^2+^ signal: −0.83 ± 0.10 and −0.51 ± 0.04 s in the light and dark phases, respectively, EMG power – Ca^2+^ signal: −0.82 ± 0.15 and −1.35 ± 0.06 s, respectively; mean ± SEM) and during the NREM sleep (lag time between peaks, EEG β power – Ca^2+^ signal: −0.31 ± 0.46 and −1.77 ± 0.39 s, respectively, EEG γ power – Ca^2+^ signal: −0.77 ± 0.18 and −1.81 ± 0.71 s, respectively; mean ± SEM; Fig. 4*f*). However, no such relationship was observed between the Ca^2+^ signal and EEG γ power during REM sleep, likely because γ power fluctuations are minimal during this state (Fig. 4*f*).

Furthermore, we focused on the averaged Ca^2+^ signal levels during each sleep-wake state, which was significantly higher during NREM and REM sleep than during wakefulness in the light phase (Fig. 4*g*). In contrast, in the dark phase, averaged Ca^2+^ signal level during REM sleep were higher than those during NREM sleep and wake states (Fig. 4*g*). Thus, we evaluated changes in the average Ca^2+^ signal level during the state transition (Fig. 4*h*). During the transition from NREM sleep to wake, the Ca^2+^ signal began to decrease in the light phase and showed a lesser tendency to decrease in the dark phase. During the transition from wake to NREM sleep, the Ca^2+^ signal began to increase in both the light and the dark phases. The Ca^2+^ signal did not change during the transition from NREM to REM sleep, and it began to decrease during the transition from REM sleep to wake in both the light and the dark phases (Fig. 4*h*). These Ca^2+^ signal pattern variations in the VLS D2-MSNs, particularly during NREM sleep induction, support the sleep-inducing function of these neurons, as indicated by our ablation experiment.

### Optogenetic activation of the VLS D2-MSNs during wake causes NREM sleep

**Figure 5.**
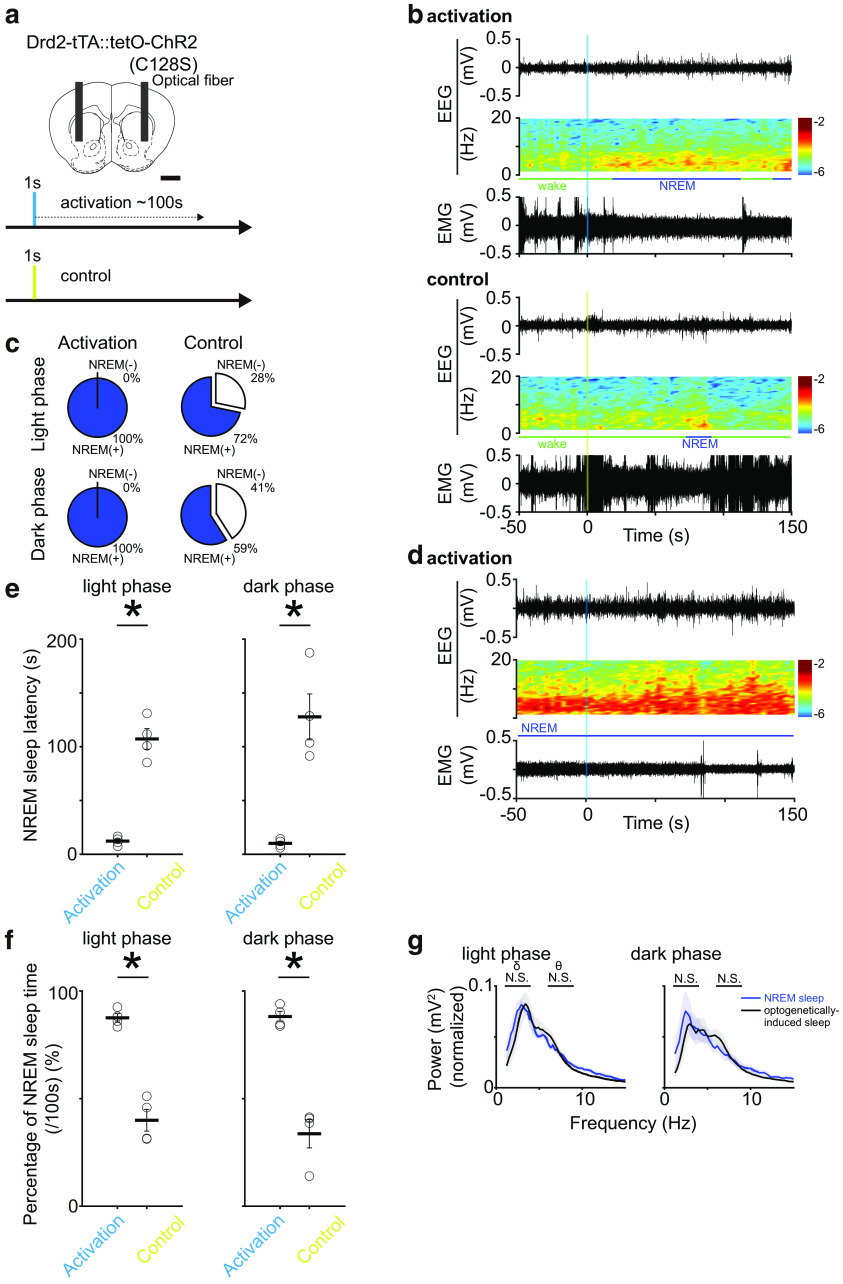
Sleep-wake state change by optogenetic activation of the VLS D2-MSNs. ***a***, Schematic illustration of optogenetic activation of VLS D2-MSNs in *Drd2-*ChR2(C128S) mice (hereafter referred to as D2-ChR2 mice). Scale bar, 1 mm. The blue and yellow shades indicate the illumination times. ***b***, Representative examples of EEG signal traces, power spectrograms, and EMG signals under the photostimulation of VLS D2-MSNs during the wake state in D2-ChR2 mice. Vertical blue and yellow shades indicate illumination times. The upper panel shows the optogenetic manipulation and the lower panel shows the control experiment. ***c***, NREM sleep induction rate which indicates whether NREM sleep occurred within 100 s after light illumination. Upper column shows a light phase and lower column shows a dark phase. In each column, left side shows the activation group (blue light illumination) and right side shows control group [yellow light illumination; *n* = 4 mice, 5–10 manipulations/recordings, a total of 49 trials for activation (light phase), 51 trials for control (light phase), a total of 38 trials for activation (dark phase), 39 trials for control (dark phase)]. ***d***, Representative examples of EEG signal trace, power spectrogram, and EMG signal under the photostimulation of VLS D2-MSNs during the NREM sleep in D2-ChR2 mouse. Vertical blue shade indicates illumination times. ***e***, NREM sleep latency after the initiation of optogenetic activation of VLS D2-MSNs during the wake state. The columns on the left and right show the light and dark phases, respectively. In each column, left bar shows the activation group, right bar shows the control group [*n* = 4 mice, 5–10 manipulations/recordings, a total of 49 trials for activation (light phase), 51 trials for control (light phase), a total of 38 trials for activation (dark phase), 39 trials for control (dark phase); *p*_light_ = 6.9 × 10^−5^, *p*_dark_ = 1.5 × 10^−3^, independent *t* test]. ***f***, Percentage of NREM sleep time for 100 s after the initiation of VLS D2-MSNs photoactivation during the wake state. The columns on the left and right show the light and dark phases, respectively. In each column, left bar shows the activation group, light bar shows the control group [*n* = 4 mice, 5–10 manipulations/recordings, a total of 49 trials for activation (light phase), 51 trials for control (light phase), a total of 38 trials for activation (dark phase), 39 trials for control (dark phase); *p*_light_ = 1.2 × 10^−4^, *p*_dark_ = 2.4 × 10^−4^, independent *t* test]. ***g***, EEG spectrum of D2-ChR2 mice during natural NREM sleep (blue line; we extracted and averaged 200 s of NREM sleep before optogenetic activation) and optogenetically induced sleep (black line) during the light (left panel) and dark phases (right panel; *n* = 4 mice, the same mice in ***c–e***; δ: *p*_light_ = 0.63, *p*_dark_ = 0.94; θ: *p*_light_ = 0.59, *p*_dark_ = 0.75, independent *t* test). Colored shades and error bars indicate SEM; *p <* *0.05.

Our ablation study and Ca^2+^ signal recordings suggest that VLS D2-MSNs have an NREM sleep-inducing function. Therefore, to determine the causal relationship between the VLS D2-MSN activity and NREM sleep induction, we performed optogenetic activation of the VLS D2-MSNs. We used transgenic mice in which only the D2-MSNs expressed the step-function-type variant of ChR2 [D2-ChR2(C128S)] and artificially activated their VLS D2-MSNs (Fig. 5*a*). In this variant, short-term light induction cause to initiate photocurrent, and it terminated ∼100 s after light induction because the “tau off” of C128S mutant ion channel is ∼106 ± 9 s (Berndt et al., 2009). The D2-ChR2(C128S) mice received 1 s of blue light illumination to open the ChR2(C128S) in the bilateral VLS D2-MSNs or 1 s of yellow light as a control (Fig. 5*a*).

Optogenetic activation of VLS D2-MSNs during the wake state in mice always prompted changes in EEG/EMG activity and induced NREM sleep within 100 s after light illumination during both the light and dark phases; however, control light (yellow light) did not always induce these changes (Fig. 5*b*,*c*). In contrast, optogenetic activation of the VLS D2-MSNs during NREM sleep resulted in no changes in sleep-wake state of animals (Fig. 5*d*). Notably, REM sleep did not occur during the first 100 s after the onset of photostimulation, whether in NREM sleep or wake state.

The NREM sleep-induction latency by the VLS D2-MSNs photoactivation during the wake state was 12.3 ± 2.0 and 10.2 ± 1.9 s in the light and the dark phases, respectively, which was significantly shorter than those of the control light illumination [mean ± SEM, *p *=* *6.9 × 10^−5^ and *p *=* *1.5 × 10^−3^ (light and dark phases, respectively), independent *t* test; Fig. 5*e*]. The percentages of NREM sleep time during 100 s after the start of VLS D2-MSNs photoactivation were 87.6 ± 1.9% and 88.1 ± 2.4% in the light and the dark phases, respectively, which were significantly higher than that of control light illumination [mean ± SEM, *p *=* *1.2 × 10^−4^ and *p *=* *2.4 × 10^−4^ (light and dark phases, respectively), independent *t* test; Fig. 5*f*]. Furthermore, EEG δ and θ powers were at the same level between natural NREM sleep and the optogenetically induced NREM sleep during both the light and dark phases (Fig. 5*g*). These results suggest that the VLS D2-MSNs activity induced NREM sleep in mice.

### Optogenetic activation of the VLS D1-MSNs induces wakefulness from NREM sleep

**Figure 6.**
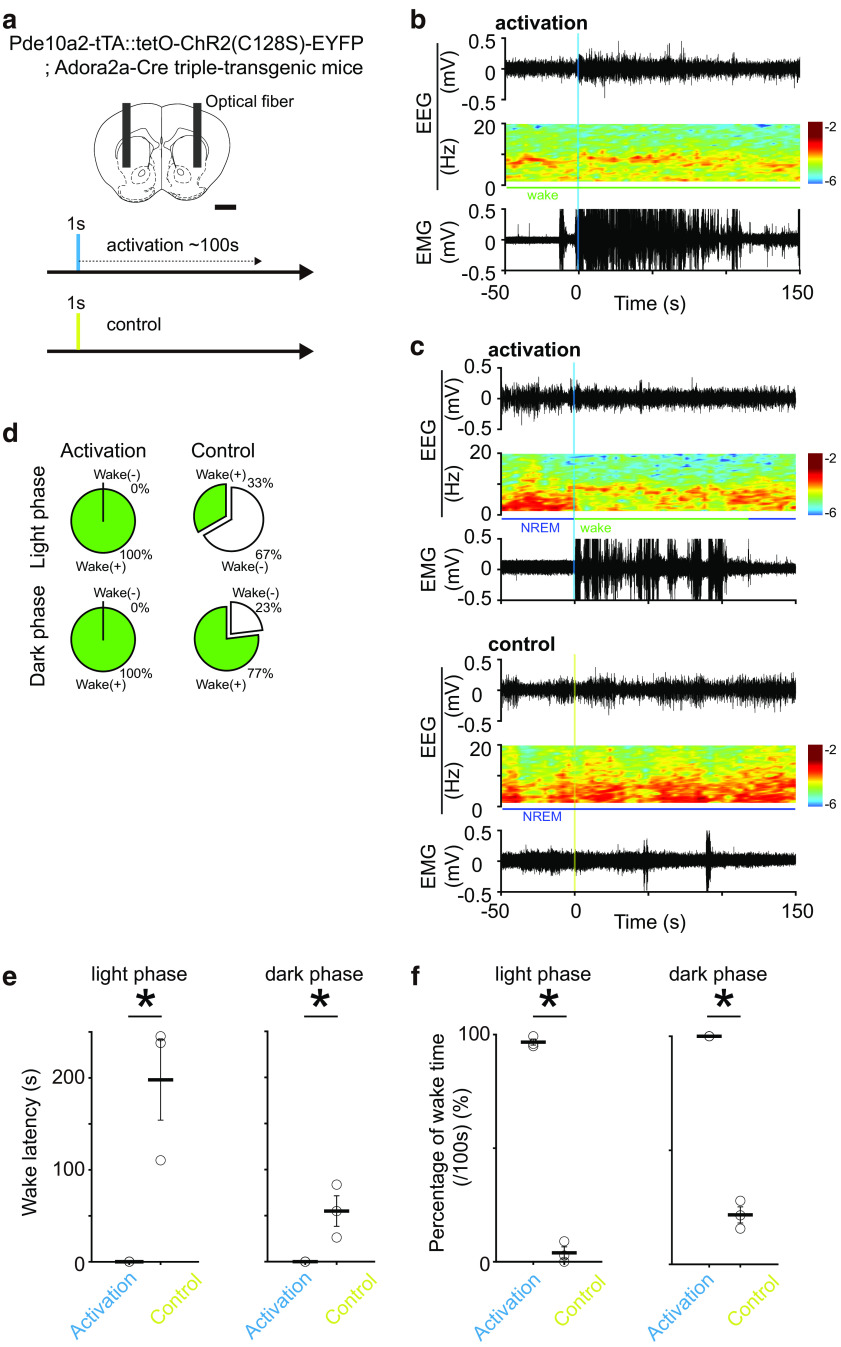
Sleep-wake state change by optogenetic activation of the VLS D1-MSNs. ***a***, Schematic illustration of optogenetic activation of VLS D1-MSNs in *Pde10a2*-tTA::tetO- ChR2(C128S)-EYFP; *Adora2a*-Cre triple-transgenic mice. Scale bar, 1 mm. The blue and yellow shades indicate the illumination times. ***b***, Representative examples of EEG signal trace, EEG power spectrogram, and EMG signal before and after optogenetic manipulation during the wake. Vertical blue shade indicates illumination times. ***c***, Representative examples of EEG signal traces, EEG power spectrograms, and EMG signals before and after optogenetic manipulation during NREM sleep. Vertical blue and yellow shades indicate illumination times. The upper panel shows the optogenetic manipulation and the lower panel shows the control experiment. ***d***, Wake state induction rate which indicates whether the wake state occurred within 100 s after light illumination. Upper column shows light phase and lower column shows dark phase. In each column, left side shows the activation group (blue light illumination) and right side shows the control group [yellow light illumination; *n* = 3 mice, 5–10 manipulations/recordings, a total of 23 trials for activation (light phase), 18 trials for control (light phase), a total of 18 trials for activation (dark phase), 20 trials for control (dark phase)]. ***e***, Wake latency after the initiation of optogenetic activation of VLS D1-MSNs during NREM sleep. The columns on the left and right show the light and dark phases, respectively. In each column, left bar shows the activation group, right bar shows the control group [*n* = 3 mice, 5–10 manipulations/recordings, a total of 23 trials for activation (light phase), 18 trials for control (light phase), a total of 23 trials for activation (dark phase), 30 trials for control (dark phase); *p*_light_ = 0.01, *p*_dark_ = 0.03. independent *t* test]. ***f***, Percentage of wake time for 100 s after the initiation of VLS D1-MSNs photoactivation. The columns on the left and right show the light and dark phases, respectively. In each column, left bar shows the activation group, light bar shows the control group [*n* = 3 mice, 5–10 manipulations/recordings, a total of 23 trials for activation (light phase), 18 trials for control (light phase), a total of 23 trials for activation (dark phase), 30 trials for control (dark phase); *p*_light_ = 6.3 × 10^−6^, *p*_dark_ = 2.4 × 10^−5^, independent *t* test]. Error bars indicate SEM; *p <* *0.05.

In the striatum, another population of MSNs, expressing dopamine receptor type 1 (D1-MSNs), is also distributed. While D2-MSNs and D1-MSNs in the dorsal striatum have opposing roles in motor control and reinforcement learning, in the VLS they have been reported to have cooperative roles in goal-directed behavior in mice (Kravitz et al., 2010; Natsubori et al., 2017). Therefore, we tried optogenetic activation of the D1-MSNs in the VLS to compare the sleep-wake regulation functions of VLS D2-MSNs and D1-MSNs. We used D1-ChR2 mice (*Pde10a2*-tTA::tetO-ChR2(C128S)-EYFP; *Adora2a*-Cre triple-transgenic mice), which harboring ChR2(C128s) only in the D1-MSNs (Fig. 6*a*). As a result, optogenetic activation of the VLS D1-MSNs during wake state did not cause any state changes (Fig. 6*b*). However, activation of the VLS D1-MSNs during NREM sleep always promptly induced wake in mice during the light and dark phases, whereas control light (yellow light) illumination did not always induce it (Fig. 6*c–f*). The wake-induction latency by the VLS D1-MSNs photoactivation was 0.15 ± 0.02 and 0 s in the light and the dark phases, respectively [mean ± SEM, *p *=* *0.01 and *p *=* *0.03 (light and dark phases, respectively), independent *t* test; Fig. 6*e*]. The percentages of wake time during 100 s after the start of VLS D1-MSNs photoactivation were 96.7 ± 1.3% and 100% in the light and the dark phases, respectively [mean ± SEM, *p *=* *6.3 × 10^−6^ and *p *=* *2.4 × 10^−5^ (light and dark phases, respectively), independent *t* test; Fig. 6*f*]. These results indicates that VLS D1-MSNs have a wake-inducing function and that VLS D2-MSNs and D1-MSNs encode opposing functions in sleep-wake regulation.

## Discussion

This study demonstrated that ablation of D2-MSN in the VLS causes an increase in the amount of wake time during the dark phase, accompanied by a decrease in sleep time. This effect becomes more pronounced as the ablation area expands to include the entire VS. Next, our fiber photometric recording revealed that the average intracellular Ca^2+^ signal level of the VLS D2-MSNs increased during the transition from wake to NREM sleep in mice and remained high during NREM and REM sleep compared with that in the wake state. Optogenetic activation of VLS D2-MSNs induced NREM sleep in mice from wake state, while VLS D1-MSN activation induced wake state from NREM sleep. These results suggest that D2-MSNs in the VLS have an NREM sleep-inducing function in coordination with those in other medial parts of the VS. Furthermore, this sleep-wake regulation function of VLS D2-MSNs was in the opposite direction to the D1-MSNs function in the same subregion.

Our ablation and optogenetic activation studies strongly suggest that D2-MSNs in the VLS have an NREM sleep-inducing function (Figs. 2 and 5), and the fiber photometric recording supports these results (Fig. 4). The VLS is distinguished from the medial parts of the VS, such as the NAc medial shell and core, based on cortical input patterns (Berendse et al., 1992; Hunnicutt et al., 2016). Several studies have demonstrated the functional specificities of D2-MSNs in the VLS, such as reward processing (Tsutsui-Kimura et al., 2017a; Yang et al., 2018; R. Chen et al., 2021). However, our findings regarding the sleep-inducing function of D2-MSNs in the VLS are consistent with those found in the medial portion of the VS (NAc medial shell/core; Satoh et al., 1999; Lazarus et al., 2011; Oishi et al., 2017; Luo et al., 2018). Furthermore, in the DTA-induced ablation experiment, we observed that the alteration of sleep/wake architecture was even more pronounced under the ablation of the D2-MSNs in the entire VS, compared with that of in the VLS (Fig. 2*b*,*c*). Therefore, the alterations in the sleep/wake pattern might be strengthened by the ablation of D2-MSNs in both the VLS and the medial part of the VS, indicating a coordinated and consistent regulation of the animal’s sleep-wake states by the D2-MSNs in these subregions of the VS. The degree of the D2-MSNs effect on the sleep/wake architecture may depend on the extent of its distribution area. D2-MSNs in the VS project to the VP (Basar et al., 2010), of which the projection from the NAc core to the VP has been reported to be involved in sleep-wake regulation (Oishi et al., 2017; Luo et al., 2018). These consistent efferent patterns could be associated with the common sleep-wake regulatory role of D2-MSNs in the medial and lateral VS.

We demonstrated that the optogenetic activation of the VLS D2-MSNs induced NREM sleep from wakefulness whereas that of the VLS D1-MSNs induced wake from NREM sleep in mice (Figs. 5 and 6). These findings strongly suggest that the VLS D1-MSNs and D2-MSNs activities themselves change the sleep-wake state of animals in the opposite direction. The reversal sleep-wake regulation function of the VLS D1-MSNs and D2-MSNs could be related to the differential dopaminergic control and/or output patterns of these two neuronal subpopulations. First, striatal D1-MSNs and D2-MSNs activities are inversely affected by the extracellular dopamine: as the D1 and D2 receptors couple with G_s_ and G_i_ proteins, respectively (Kebabian and Calne, 1979), the dopamine signal has excitatory and inhibitory effects on the activity of D1-MSNs and D2-MSNs, respectively. Thus, dopaminergic control often causes the opposing function of striatal D1-MSNs and D2-MSNs, and their balance is expressed in functional outputs such as motor controls (Cox and Witten, 2019). It could be the same for the sleep-wake regulation function of the VLS D1-MSNs and D2-MSNs. Next, the partially differential output patterns of the VS D1-MSNs and D2-MSNs might cause their reversal of sleep-wake regulation functions. D2-MSNs in the VS consistently project to the VP, while the D1-MSNs in the NAc project to not only the VP but also the ventral mesencephalon (VM; Kupchik et al., 2015; Liu et al., 2022). D1-MSNs in the NAc core have a wake-promoting role via the VM and lateral hypothalamus (Luo et al., 2018) and artificial inhibition of GABAergic neurons in the VM increases wakefulness time (Takata et al., 2018), whereas the VS D2-MSNs projection to the VP could involve NREM sleep induction (Oishi et al., 2017; Luo et al., 2018). These findings suggest that the VLS D2-MSNs activation could induce NREM sleep only through the VP whereas the VLS D1-MSNs activation-induced wake could be mediated by the inhibition of VM neurons.

NREM-sleep inducing function of the D2-MSNs and opposing wake inducing function of the D1-MSNs that we demonstrated in the VLS are consistent with D1-/D2-MSNs functions in the NAc core region (Oishi et al., 2017; Luo et al., 2018). The common reciprocal sleep-wake regulation function of D1-MSNs and D2-MSNs across the VS subregion may aid in the identification of sleep-wake regulation neuronal circuits centered on the VS D1-MSNs/D2-MSNs. As previously mentioned, since the dopaminergic input from the SNr to the entire VS has a reverse effect on D1-MSNs and D2-MSNs (Scofield et al., 2016), it may be involved in the sleep-wake regulation function. Meanwhile, the glutamatergic inputs from the distinct cortical area to each VS subregion (Berendse et al., 1992; Hunnicutt et al., 2016) may have consistent effects on the MSNs if they participate in sleep-wake regulatory mechanisms. In turn, the common output of VS D1-MSNs and D2-MSNs to the VM and VP, respectively, could be engaged for sleep-wake regulation (Basar et al., 2010; Kupchik et al., 2015; Liu et al., 2022). Additionally, the latency to sleep/wake transition differs between D1 or D2-MSN stimulation (Figs. 5 and 6). Even in the natural sleep-wake cycles of animals, the transition from sleep to wakefulness occurs rapidly, whereas the transition from wakefulness to sleep is more gradual (Saper et al., 2010). Differences in the effects of each sleep-wake inducing neuronal activity on the entire cortex may be responsible for the differences in sleep/wake transition latency.

Our fiber photometric recordings revealed that the compound intracellular Ca^2+^ signal in the VLS D2-MSNs showed the sleep-wake state-dependent variation and the mean signal level was elevated during the wake-to-NREM sleep transition both in the light and dark phases (Fig. 4). These findings support our conclusion of the sleep-inducing function of the VLS D2-MSNs derived from our ablation and optogenetic experiments (Figs. 2 and 5). The VLS D2-MSNs activity can be affected by dopaminergic input from the ventral tegmental area (VTA; Mingote et al., 2019) and glutamatergic input from the insular cortex (Berendse et al., 1992; Hunnicutt et al., 2016). The activity of VTA dopaminergic neurons varies depending on the sleep-wake state, which is higher during wakefulness and REM sleep and lower during NREM sleep (Eban-Rothschild et al., 2016). Therefore, VTA dopaminergic inputs, which could suppress D2-MSNs activity via the Gi-coupled D2 receptor, could cause the VLS D2-MSNs activity patterns to be lower during wakefulness and higher during NREM sleep. However, the higher activity of VLS D2-MSNs during REM sleep, particularly in the dark phase (Fig. 4*g*), cannot be explained by variations in the dopaminergic input. The increase in the Ca^2+^ signal level of the VLS D2-MSNs during REM sleep may be influenced by glutamatergic neuronal input from the insular cortex. A previous study showed that lesions in the insular cortex led to decreased wake time and increased NREM and REM sleep times (M. Chen et al., 2016). The fluctuation of neuronal activity in the insular cortex across sleep-wake states is currently unclear; however, given its potential involvement in sleep/wake regulation, it is not unexpected that glutamatergic neuronal projections from the insular cortex may impact the activity of D2-MSNs in the VLS across different sleep-wake states. In addition, these diverse state-dependent neuronal inputs might cause detailed differences in the VLS D2-MSNs Ca^2+^ signal fluctuations between each sleep-wake state in terms of their relationship with EEG/EMG activity in mice (Fig. 4*e*,*f*). Further studies are required to elucidate the sleep-inducing functions of the VLS D2-MSNs at the neuronal circuit level.

We observed that the change in sleep/wake architecture caused by VLS/VS D2-MSNs ablation occurred only during the dark phase (Fig. 2). Previous studies also reported that the inhibition D2-MSNs in the NAc core induced wake state in mice during the dark phase, although they did not mention during the light phase (Oishi et al., 2017; Luo et al., 2018). Meanwhile, Ca^2+^ signals in the VLS D2-MSNs increased during the transition from wake to NREM sleep in both light and dark phases (Fig. 4). These suggest that the VLS D2-MSNs increase their activity at the onset of NREM sleep in both the light and dark phases; however, this activity might exert its sleep-inducing function during the dark phase. Besides, we observed that optogenetic activation of VLS D2-MSNs and D1-MSNs promptly induced sleep-wake state change regardless of the light or dark phases (Figs. 5 and 6). These findings suggest that the firing activity of D1/D2-MSNs has a potential role in sleep-wake state change regardless of the light/dark phases, however, there might be a time-dependent gating mechanism for VLS D1/D2-MSNs output for sleep-wake regulation.

Our study has two limitations. First, the D2-MSN ablation method in D2-DTA mice (Tsutsui-Kimura et al., 2017b) cannot evaluate the sleep-wake regulatory function of D2-MSNs in each subregion of the VS separately, except for the VLS. Several prior studies demonstrate a causal relationship between the activity of the D2-MSNs in the medial part of the VS, particularly the NAc core, and NREM-sleep-inducing function, and these findings complement our result (Oishi et al., 2017; Luo et al., 2018). Next, it has been pointed out that fluorescent probes expressed in the living brain and detected by the fiber photometry system could be affected by cerebral blood volume (Ikoma et al., 2023a, b). The evaluation of YC signal fluctuations particularly during REM sleep should be cautious because cerebral blood flow is increased (Braun et al., 1997; Tsai et al., 2021; Ikoma et al., 2023b). The proposed new fiber photometry setup and analysis method to exclude the effects of cerebral blood flow (Ikoma et al., 2023a, b) could improve the accuracy of our neuronal activity assessment based on the YC signal measurement in the future.

In conclusion, our results demonstrate that D2-MSNs in the VLS are essential for the maintenance of sleep/wake architecture and have an NREM sleep-inducing function.

## Synthesis

Reviewing Editor: Christopher Colwell, UCLA School of Medicine Decisions are customarily a result of the Reviewing Editor and the peer reviewers coming together and discussing their recommendations until a consensus is reached. When revisions are invited, a fact-based synthesis statement explaining their decision and outlining what is needed to prepare a revision will be listed below. The following reviewer(s) agreed to reveal their identity: NONE. Note: If this manuscript was transferred from JNeurosci and a decision was made to accept the manuscript without peer review, a brief statement to this effect will instead be what is listed below.

This is an interesting manuscript that identifies new information about the role of striatal D2 neurons in sleep. Many of the reviewer concerns could be addressed via revision. The critical concern that the manipulations were not selective to a specific ventral striatal subregion does not reduce the more general interest that could follow from the simpler conclusion that D2-MSNs are important in sleep/wake regulation.

Reviewer 1

The authors use Drd2-tTA::tetO-DTA mice to ablate D2-expressing cells. They say that this “is initiated specifically in the VLS and follows spatial progress within the ventral striatum (VS) in doxycycline (DOX)-dependent manner” (lines 288-290). They then discuss progressive hypofunction/dysfunction of the VLS/VS. The concern is that they present no data to support this progression. The authors should use a more targeted approach to ablate D2 cells in the VLS.

The in-situ hybridization data in Figure 1d is not convincing evidence of hypofunction and death of D2 cells as concluded by the authors. A colorimetric reaction has limited quantitative power, especially given the lack of controls. A more direct measure of hypofunction or cell death is necessary.

The authors describe doing EEG recordings relative to Dox, but it is not clear whether mice are housed continually in the recording chamber and tethered or how long they are in the chamber prior to recording. This is important as their behavior will change as they acclimate to their new environment. It would also be helpful to have controls for these experiments to show that other factors like acclimation, change in diet, etc are not influencing their data.

There is a concern with the sleep/wake cycle data. Mice typically show a robust diurnal variation in sleep/wake distribution (more sleep during the day and less at night). However, in the data shown in Figure 2, the 24hr sleep wake cycle is essentially absent at baseline (day -1) whereas the day 10 post DTA looks more like what one would expect for 24hr sleep/wake distribution.

The ablation data would suggest that D2 cells are impacting sleep/wake during the dark phase, but the authors only conducted the photometry experiment during the light phase. Given the diurnal variation in striatal neuron activity and their phase-specific effect, it would be helpful to have Ca2+ dynamic from both the light and dark phases.

The latency to sleep/wake transition is different in D1 vs D2 stimulation (Figures 5-6). This seems like more than a simple opposing effect described in the manuscript.

The authors discuss other studies that have previously examined the role of the ventral striatum, but this discussion does not seem complete. For instance, the authors discuss that the ventral medial striatum’s role in sleep/wake regulation has been established yet the role of the ventral lateral striatum is unknown. The strategies used in Oishi et al 2017 and Luo et al 2018 targeted both medial and lateral NAc and showed that D2-MSN activation induces sleep (and D1 and D2 MSNs have opposing roles in modulating sleep/wake in the case of Luo et al). There needs to be a more thoughtful discussion of how the work presented in this manuscript advances our understanding of ventral striatal control of sleep/wake in the context of existing work.

The authors use parametric tests like t-tests but there is no discussion of whether/how data were evaluated to ensure that assumptions for these tests were met.

Minor comments

Figure 1e would fit better in Figure 2.

Reviewer 2

This study by Kato et al. aimed to understand the role of D2-MSNs in the ventral lateral striatum in regulating sleep-wake states, comparing it to the previously established role of D2-MSNs in the medial ventral striatum. They presented layers of evidence that VLS D2-MSNs have an NREM sleep-inducing function in coordination with those in the medial ventral striatum. The study was laid out strategically, addressing loss of function, gain of function, as well as state-associated changes. The combined usage of transgenic mouse lines, viral mediated gene transfer, in vivo fiber photometry, in vivo optogenetics, and simultaneous EEG recordings represents a state-of-the-art approach. The results were robust. The conclusion was supported by the results and the interpretations were appropriate. The narrative was clear with sufficient experimental details in methods and analyses.

A few moderate to minor concerns are as follows:

(Moderate) The results showed VLS D2-MSN’s potential role in regulating REM sleep (Fig. 2b, DOX-off day 7), and that D2-MSN activities were high during REM sleep (Fig. 3g), however, potential causal relationship was not tested (or not reported) in the following optogenetic experiments. For example, whether there were changes in REM sleep following direct stimulation of D2-MSNs was not reported.

(Moderate) Related, it was not shown what the effects may be following acute inhibition of VLS D2-MSNs during NREM or REM sleep.

(Minor) From Fig 2 it was concluded that “ablation of D2-MSN in the VLS causes an increase in .. wake time during the dark phase, accompanied by a decrease in sleep time.” This may be misleading as a decrease in dark-phase NREM sleep was not yet significant at DOX-off day 7, when D2-MSNs were fully ablated in the VLS. NREM sleep loss became significant by DOX-off day 10, suggesting the involvement of entire VS.

(Minor) Fig. 2c, 2e, 2g, and Fig 3 statistics: It may be more appropriate to use 2-way repeated measure ANOVA rather than paired t-tests.

(Minor) For readers not familiar with genetics, there needs to be a brief explanation on why “Pde10a2-tTA::tetO- ChR2(C128S)-EYFP; Adora2a-Cre triple-transgenic mice” is essentially D1-ChR2 mice.
