# Erratum: Kato et al., “Dopamine Receptor Type 2-Expressing Medium Spiny Neurons in the Ventral Lateral Striatum Have a Non-REM Sleep-Induce Function”

**DOI:** 10.1523/ENEURO.0468-23.2023

**Published:** 2023-12-08

In the article “Dopamine Receptor Type 2-Expressing Medium Spiny Neurons in the Ventral Lateral Striatum Have a Non-REM Sleep-Induce Function,” by Tomonobu Kato, Kenji F. Tanaka, and Akiyo Natsubori, which published online September 13, 2023, a reference was omitted. In the Animals subsection of the Materials and Methods, the sentence “D1-ChR2 mice (*Pde10a2*-tTA::tetO-ChR2(C128S)-EYFP; *Adora2a*-Cre triple-transgenic mice) were obtained by crossing *Pde10a2*-tTA mice, tetO- ChR2(C128S)-EYFP mice, and *Adora2a*-Cre mice.” should have included a reference to Durieux et al., 2009 at the end. The full omitted reference appears below, and the online version has been corrected.
